# Supplementary material for: Dynalign II: common secondary structure prediction for RNA homologs with domain insertions
Source: Nucleic Acids Res. 2014 Nov 21;42(22):13939–48. doi: 10.1093/nar/gku1172 (PMC4267632; doi:10.1093/nar/gku1172)
Supplement: SUPPLEMENTARY DATA [file supp_gku1172_nar-02021-z-2014-File012.zip › manual/javadoc/overview-frame.html]

Overview List


All Classes

## Packages

- RNAstructure\_java\_drawing.source
- RNAstructure\_java\_drawing.source.dialogs
- RNAstructure\_java\_drawing.source.menus
- RNAstructure\_java\_drawing.source.proxy
- RNAstructure\_java\_drawing.source.runners
- RNAstructure\_java\_interface.source
- RNAstructure\_java\_interface.source.menus
- RNAstructure\_java\_interface.source.startup
- RNAstructure\_java\_interface.source.windows
- RNAstructure\_java\_utilities
